# Family and friends: Supporting oral care in care homes

**DOI:** 10.1111/ger.12404

**Published:** 2019-04-17

**Authors:** Rhiannon J. Jones, Ilona G. Johnson, Maria Z. Morgan

**Affiliations:** ^1^ Department of Education, Scholarship and Innovation (DESI) Cardiff University School of Dentistry Cardiff UK; ^2^ Applied Clinical and Public Health Research Cardiff University School of Dentistry Cardiff UK

**Keywords:** dental care, elders, family, nursing homes

## Abstract

**Objective:**

To consider the role of family and friends in supporting oral care.

**Background:**

People who live in care homes are susceptible to oral health problems, which can be detrimental to their health and personal and social well‐being. External support from family members and friends has been indicated as being important for maintaining oral health for this vulnerable group of care home residents.

**Materials and methods:**

Qualitative one‐to‐one interviews were undertaken with care home residents, in Cardiff, UK. Further interviews were undertaken with care home personnel with responsibility for oral health care in order to contextualise residents’ interview data. Interviews were audio recorded, transcribed and analysed using a thematic approach.

**Results:**

A total of 26 interviews were conducted with care home residents and four interviews with care home personnel, across five care homes. Three main themes emanated from the data relating to co‐supporting oral care: supplying oral care products; accessing dental care and enabling self‐management of oral care problems. There were no spouse caregivers; family and friends acted as co‐supporters of oral care providing a link to residents’ pre‐care home lives by informing the care home personnel of their relatives’ normal routines. An overarching theme “balancing roles – maintaining the equilibrium” emerged from the data reflecting the roles that both care home personnel and family and friends had in balancing the needs, care and well‐being of the resident.

**Conclusion:**

This study suggests that there are opportunities to improve oral health by providing support for family and friends of those people who are living in care, especially in relation to supplying oral care products, enabling self‐management of oral care problems and accessing dental care.

## 1 INTRODUCTION

Good oral health is important for care home residents’ general health, nutritional intake^1^, ^2^, ^3^, ^4^ and well‐being.^5^ The world population is ageing and people are living with increasingly complex health conditions. The number of people who live in care homes has remained relatively stable over recent years,^6^ but the complexity of health issues has increased. Most residents take a number of medications, many of which are associated with xerostomia (dry mouth).^7^, ^8^ Also, as evidenced by the most recent UK Adult Dental Health Surveys (ADHS), adults are now keeping some of their natural teeth into old age.^9^, ^10^, ^11^ As a consequence, people who live in care homes are particularly susceptible to oral health problems,^7^ which in turn can be detrimental to their general health and their personal and social well‐being.^10^ Good oral health is therefore important for maintaining the quality of life and health of older people who are living in care. Surveys indicate that most care home residents carry out their own oral care.^12^

Care systems often rely on informal care arrangements: a review in 2006 indicated that between 3.4 and 4 million people in England were providing informal care to people over the age of 65.^13^ Women are more likely than men to provide informal care throughout most of the life course.^14^ While there is evidence to suggest that the support of informal carers can be of benefit to residents and improve well‐being,^15^ there is relatively little information about the most effective ways that informal caregivers can support care.^16^, ^17^ Few studies have considered the role of informal caregivers in supporting oral health care in care homes, however, a small number of qualitative studies in Australia and Canada have identified external support from family members and friends as being important for maintaining oral health for this vulnerable group.^18^, ^19^, ^20^

The NICE guidelines for “Oral health for adults in care homes” [NG48] include recommendations which relate to care home policies on providing residents with support to access dental services. Recommendation 1.1.6 specifically highlights that care home “residents and their families or friends (need to be made aware) of care home policies to promote health and well‐being, including mouth care.”^21^ Furthermore, one of the systematic reviews underpinning the recommendations indicated that there was a small amount of evidence showing that friends and family could provide positive support for oral care.^21^

This study explored care home residents’ views of dental care in care homes. This analysis focusses on the role of family and friends in supporting oral care.

## 2 METHOD

Approval for the study was given by the Cardiff University, Dental School Research Ethics Committee. In addition, advice was sought from the UK National Research Ethics Service who advised that no additional approvals were required.

Care homes in Cardiff, the Welsh capital with a population of 341 000, were identified using Care and Social Services Inspectorate Wales (CSSIW) care home inspection reports. Care homes for young people with learning disabilities and for those with severe mental health problems (who would not be able to consent to participate in the study) were excluded. Eligible care homes were then contacted and the researcher scheduled a date to visit for the interviews to take place. Residents were purposively sampled, based on the care home manager's identification of residents who were deemed able to consent and to be “suitable.” “Suitable” in this context refers to those residents who had the mental capacity to understand that they were agreeing to take part in a study which was voluntary; residents needed to be able to have a lucid conversation with a member of the research team in relation to oral care. Residents were then approached by the researcher and were given verbal and written information about the study and asked if they would like to take part. At this point, consent was obtained if the researcher felt the resident had the capacity to give informed consent.

**Figure 1:**
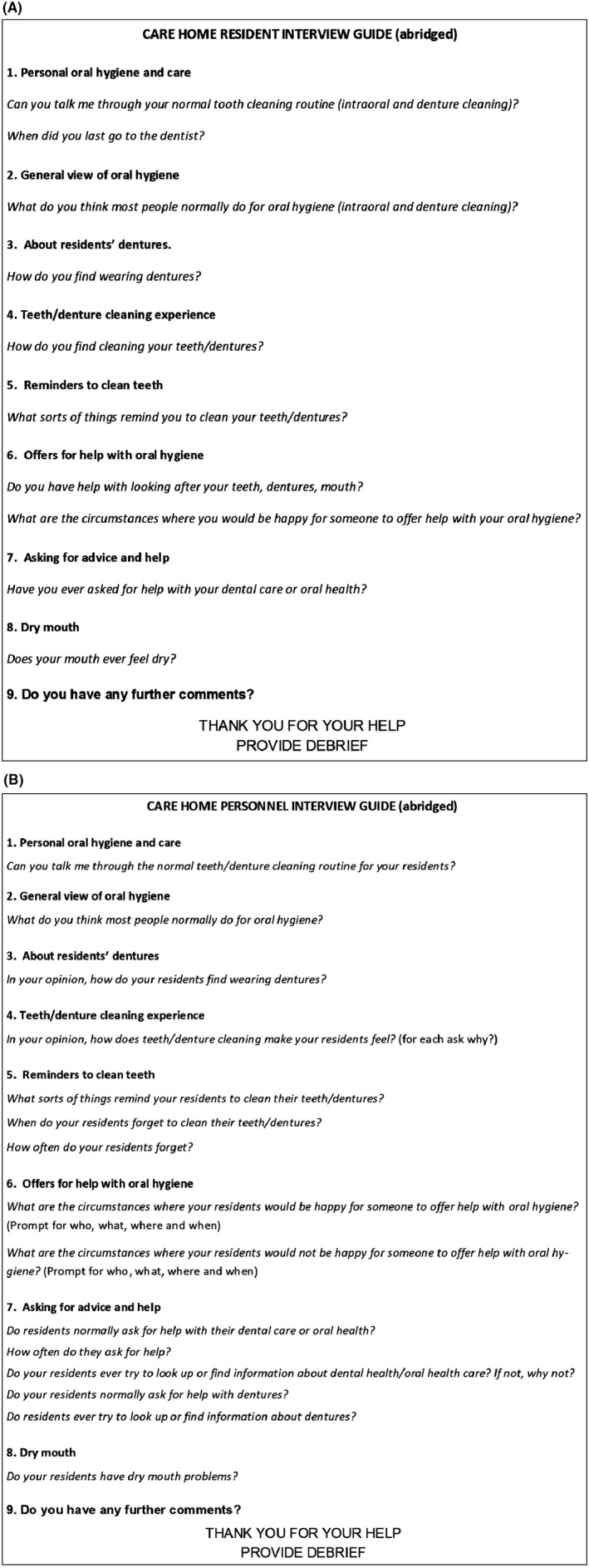
Abridged interview guide. A, Care home residents. B, Care home personnel

Data were collected using semi‐structured one‐to‐one qualitative interviews with care home residents (Figure 1A), and further interviews were undertaken with care home personnel with responsibility for oral health care to contextualise residents interview data (Figure 1B). All interviews were audio recorded, transcribed and analysed using a thematic approach to data. Analysis was assisted by NVivo 10 software. Data collection were completed when no new themes emerged from the interviews with residents.

### 2.1 Data collection—residents

As the majority of the residents were vulnerable and often needed physical assistance, it was decided that two members of the research team would be present for the interviews. Interviews were conducted in a quiet location in the care home, often in the residents’ own private room. One research team member led the interview with the other listening, making notes and interjecting when required. An interview guide was used to help guide the interviews but the residents were also allowed to “chat” as the conversation developed. Interviews were audio recorded, and manual notes were also taken. Residents agreeing to participate in the study were interviewed on the same day as consent was given in order to minimise disruption for the care homes.

### 2.2 Data collection—care home personnel

Arrangements were made to interview care home personnel who had agreed to participate in the study. Interviews were either conducted in the care homes or by telephone with a single researcher. Interviews were audio recorded, manual notes were taken and each interview ended with a debrief, with the option to add or clarify comments.

## 3 RESULTS

A total of 14 care homes from across Cardiff were identified. After initial discussions with care home managers, six homes were deemed unsuitable for the study as the residents were unable to consent to participate. One home agreed to participate, but later withdrew because of concern that their residents lacked the mental capacity to take part and a further two care homes declined to participate. In total five care homes, one nursing and four residential, took part in the study. The data collection process culminated in 26 interviews with care home residents and four interviews with care home personnel.

The care home personnel interviewees came from four of the five care homes, an interview was unable to be scheduled with a staff member from the fifth home because of care home time pressures. All four care home personnel were responsible for oral health but had different duties within the cares home settings; one was a care home manager, another was a team manager and two were carers.

**Figure 2:**
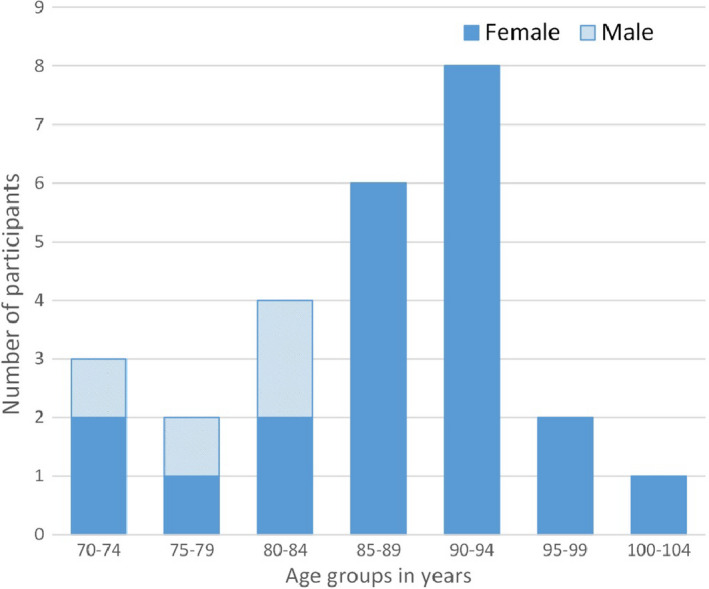
Age distribution of care home resident interviewees [Colour figure can be viewed at http://www.wileyonlinelibrary.com]

The majority of the interviewees were female with only four male residents taking part in the study (Table 1). This reflects the UK care home population with a greater number of women in care homes compared with men.^22^ The age distribution of study participants is presented in Figure 2; almost two‐thirds of the residents were aged 85 and over (17/26, 65%). All the interviewees were permanent care home residents (no respite), with varying degrees of mental capacity. One care home did not take dementia patients while the remaining homes had a mix of residents with physical and mental challenges. All interviewees were deemed to have appropriate capacity to take part.

**Table 1:** Demographic characteristics of care home resident interviewees

	Age in years	
Resident's age		
Range	70.0	100.0
Mean	86.6	
SD	7.9	

	n	%
Resident's gender		
Female	22	84.6
Male	4	15.4
Care home		
Nursing	7	26.9
Residential	19	73.1

In this study, there were no spouse caregivers and the majority of residents were supported by their children, notably daughters. However, sons, cousins and friends were also mentioned. Family and friends acted as co‐supporters of oral care providing a link to residents’ pre‐care home lives by informing the care home of their relatives’ normal routines.

### 3.1 Family and friends—co‐supporters of oral care

#### 3.1.1 Roles

All residents reported that they carried out their own intraoral hygiene care, none received help with this from care home personnel or family/friends. Although there was no reported physical assistance with brushing, poor oral hygiene was observed on more than one occasion by the research team. However, some residents received prompting from care home personnel to clean their teeth/dentures, which sometimes included care home personnel demonstrating brushing techniques, through role play, using hand gestures for the residents to follow. Some also received assistance with extraoral care tasks, for example denture cleaning. For most residents, family and friends took significant support roles for oral health. Three main themes emanated from the data relating to co‐supporting oral care, these were: supplying oral care products, enabling self‐management of oral care problems and accessing dental care.

#### 3.1.2 Supplying oral care products

Care home personnel were asked about how the residents obtained their oral care products. In the vast majority of cases the care homes “operated” on the premise that families would be involved in the provision of “hygiene” products.

they [residents] provide all the toiletries and if they run out matron always has a stock in the office spare. Whenever anyone runs out we can supply them. [Care Home Staff Member]

Oral care products such as toothpaste, toothbrushes and denture care products were perceived as part of routine hygiene toiletries and were discussed in the same context as soap, shampoo etc as opposed to healthcare products. There was an expectation that these “hygiene” products would normally be provided by family and friends. The care home would only provide these if necessary as a backup. This was echoed in the interviews with the residents while discussing the provision of oral care products.

No I get my own, no I get my daughter to [buy it] [Resident]

‐‐‐‐‐‐‐‐‐‐‐‐‐‐‐‐‐‐

Well the care home will buy, either go and buy it for you, but mostly my daughter does my shopping for me. [Resident]

Family/friends enabled residents to access products available outside of the care home allowing them to indicate personal choice and preference. This also provided a link to their pre‐care home life.

Right, we have a shop here that we purchase toothpaste off. Um, and there’s also the families, some of the families bring in their own items for their relatives cause, you know what they’re like, they might be stuck in their way, [for example] they might always use Colgate. [Care Home Staff Member]

Additionally, family and friends sometimes made choices on the resident's behalf, believing it would benefit their oral care.

I think my daughter bought it [electric toothbrush], for me, I’m not sure, I’m not at all sure if it was my daughter or my husband…I think they thought it would be nice for me. [Resident]

#### 3.1.3 Enabling self‐management of oral care problems

Residents described undertaking self‐help behaviours for oral health problems, and this self‐management was often supported by family and friends. Residents often sought advice from visitors before (or instead of) the care home personnel, or seeking expert opinion. The main reasons conveyed for this were residents concern for carers’ busy schedules and not wishing to burden “their” carers with additional work and also because of embarrassment and pride.

Well I’ve got a little container, you know. Which I’ve got but I’m afraid I don’t use, mainly because there are night staff here and if you want to call the night staff, well you want to be able to speak distinctly and not have anything else to think about.

You said you want to be able to speak to the night staff. Would you mind them seeing you without your teeth..?

I’d prefer to be seen with them really

… So that’s why you sleep with them then?

Yes, I suppose it is the main reason. Yes I’ve got to admit to that….Vanity it is, isn’t it? [Resident]

This theme related to residents sharing information with their family and friends, as they were often chosen as confidants for health problems, including oral health.

Residents described how their friends and family often took the initiative to help deal with oral health problems. As illustrated below, one resident confided in a friend about an oral problem. As a result, the friend selected and purchased a product to “help” treat the oral condition.

Do you use a mouth rinse at all?

Oh, I had one, I’ve just suddenly developed em. Oh you’ve spotted it….[points to mouthwash]…it’s not been used, only once. I showed my friend last week when she was visiting me, my tongue which was I think even worse than it is now [shows tongue]. And she immediately went out and bought me that, but I tried it and it burnt my mouth. It’s not a bit comfortable to use. [Resident]

In this example, the mouthwash exaggerated the problem and resulted in the care home contacting a General Medical Practitioner to visit the resident. This demonstrates how family and friends enable the self‐management of oral problems but also highlights the potential risk of lay people's advice.

#### 3.1.4 Accessing dental care and dependency for access

Not all residents were “registered” with a general dental practitioner. Some of the residents, who were physically able to do so, described how they booked dental appointments for themselves and arranged to be taken there. For others, family and friends provided a link to general dental practices. Family and friends booked dental appointments for residents and transported them to dental service providers for treatment. Among family members daughters were most often reported as providing this support.

I started with a new dentist when I came here because my old dentist in Church Road was retiring and you know anyway it would have been inconvenient as I would have had to climb narrow stairs to get there and I can’t climb any stairs let alone steep narrow ones so I enrolled in the one [dental practice] at the bottom of Dunroamin Road.

And the home helped organise that for you?

No, I don’t think they did. I did that, a friend took me there and I otherwise dealt with them on the phone and I told the home when I had an appointment and that my friend was taking me and it just went on like that. They’ve [the care home] not really had any input into that. [Resident]

Some of the residents without family support said that they had attended the dentist regularly in the past, but since coming into care, over time, this had reduced or stopped completely as a result of declining health and an inability to access dental services. Furthermore, some of the residents in the study had asked for dental appointments but found when they did, that appointments were not forthcoming. As such, they did not bother asking any more. However, residents did indicate that they would ask for an appointment if they were in pain.

Is that something you would like to do

Would you like to see a dentist?

I’d like to have one call, you know see regular. When I say regular I mean quite a distance apart but at regular times just to check because I’ve got no idea if my teeth are still active.

You’re not getting pain?

No I’m not getting pain.

But you wouldn’t mind getting a quick check up?

Oh no [positive response].

Would that be something you’d ask the girls [carers] to sort out for you?

No. Every time I ask, which is not very often, the carers say “we’ll tell so and so, matron or so and so”, and that’s the end of it. [Resident]

### 3.2 Maintaining the equilibrium

**Figure 3:**
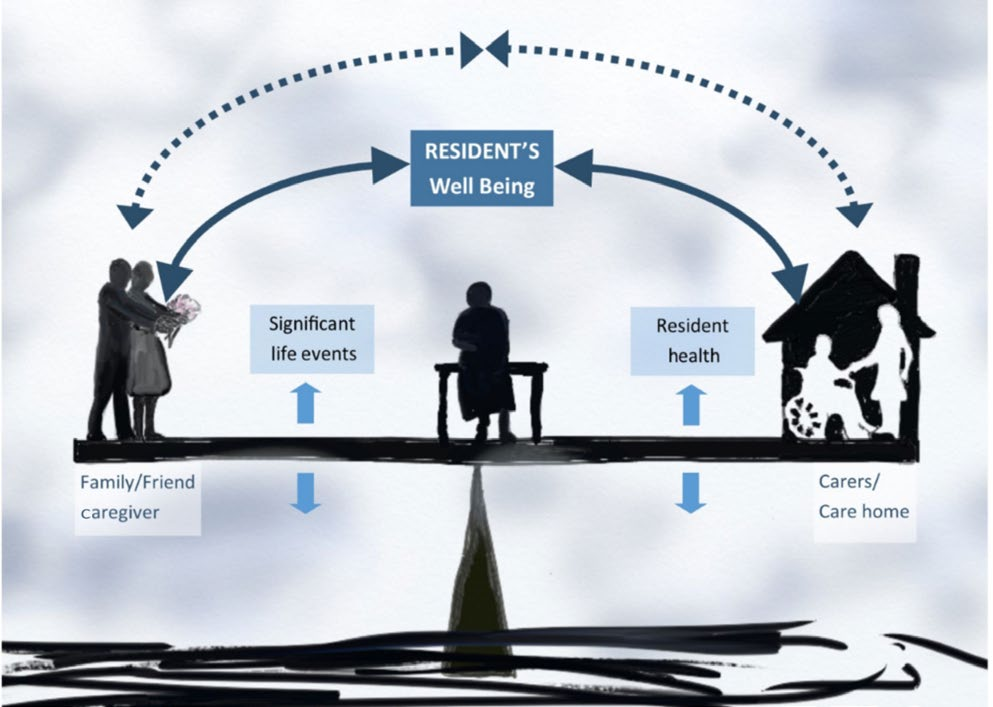
Balancing roles—Maintaining the equilibrium—a diagrammatic representation of the overarching theme, reflecting the roles that both care home personnel and family and friends have in balancing the needs, care and well‐being of the care home resident [Colour figure can be viewed at http://www.wileyonlinelibrary.com]

Family and friends supported the resident in many ways; those of which related to oral care have been discussed. An overarching theme “balancing roles – maintaining the equilibrium” emerged from the data reflecting the roles that both care home personnel and family and friends had in balancing the needs, care and well‐being of the resident. The data suggested that there was an intrinsic balance in the relationship between resident, care home personnel and informal (family/friend) caregiver (Figure 3). This interaction was individualised to each resident and varied over time.

Individual roles in delivering care varied depending on perceived “dependency” of the resident and their relationship(s) with family and friends. Some residents were strongly involved in directing their own care, as in the case of the resident below, while others were significantly reliant on others.

You obviously go to your dentist regularly, as you’ve said twice a year?

And I’ve started with the hygienist as well, they didn’t offer it. The dentist didn’t suggest it or that, and I’d been used to going every time I had a dental inspection at my old dentist, I was used to having a half hour with the hygienist first. They arranged it specially because getting there was a bit of a problem for me and they didn’t want me to do two journeys when one would do, so that worked out well and I’m going to ask for that in the new dentist next time I make an appointment. [Resident]

Responsibilities shifted between the different people involved in care at different times in an attempt to maintain the balance of well‐being and health for the resident (Figure 3). Variations in general or oral health of the resident and significant life events of informal caregivers (family members, friends) had an impact on this balance, which affected the care of the resident. This is reflected in one resident's account below.

They’re [teeth] not perfect now because I feel, during the time I’ve moved in [to the care home], I feel I’ve not done justice to my teeth really. I don’t know, maybe, it’s sort of that I’ve not spent as much time cleaning them or something. But I know really I should be going to him [dentist] now again and I’m waiting really you know, for the appointment because I have to depend, you see, on other people taking me and what my son he always took me you know, of course, unfortunately he had a stroke and it’s thrown everything. It’s thrown all the appointments because my daughter in law she’s been having to fill in all kinds of things that he used to do for me, you see as well. It’s made it very, very awkward. So I’m waiting at the moment, you know to go, cos I know I need some treatment. And it’s a shame after all this time now that if I’m going to lose my teeth or something like that, you know, because of things happening. [Resident]

### 3.3 Residents without family support

#### Blurred lines, changing roles

Care home personnel often relied on family and friends as informal carers to support care. At times, when residents have no informal carer to provide additional support, some care home personnel adopted this role. When this occurred carers often acted well above and beyond the role of a paid “carer.” For example:

One carer explained how some of the staff felt they could not ask families to buy personal care products for residents and instead used their own money for this. Care home personnel often demonstrated a close bond to particular residents and brought in personal care “treats” for them.

Does the home provide any products at all?

No we don’t. Unless for example me…… if I win on the lottery £5 or £6 I usually buy soap or toothpaste or shavers, because it’s sometimes it’s hard to ask the families to bring stuff in. So me and one of the other staff are the ones who are doing that really, we buy it for them. [Care Home Staff Member]

Residents and carers described how they both developed a sense of friendship with each other in the care home environment. This was echoed by some residents who reported having a “chosen” carer. Those with a preferred carer would prioritise talking to that carer to report problems. In this way, some of the care home personnel were helping to maintain the equilibrium, for those without family and friends support, by taking on the role of an informal (family/friend) caregiver.

## 4 DISCUSSION

Unusually, this study focussed on the experiences of care home residents whereas the majority of previous research has investigated the experiences of care home personnel and family members. The primary aim of this study was to investigate views of oral care among care home residents. One theme emanating from the data was the role of family and friends in caring for relatives during their time in residential care. Many family and friends were actively involved in supporting care for residents. This is consistent with reports of informal support in care research of oral care in care homes.^13^, ^18^, ^19^, ^20^

This study identified that the role of the residents’ family members and close friends often went beyond providing companionship during visits. This may explain why informal caregivers can assist and improve well‐being among residents.^15^ In the present study, informal caregivers supported the resident in a number of ways and participated in enabling or providing “care” for the resident. Most often, informal carers provided products for teeth and a means of making appointments and getting to dental care, similar to the level of support also identified in a small number of studies in Canada and Australia.^18^, ^19^, ^20^

The present study highlights the extent to which family and friends were regularly involved in supplying personal care products for most residents. Dental products in particular were viewed by both residents and care home personnel as “toiletries” and not necessarily under the umbrella of healthcare products. Product selection was sometimes guided by the resident but for others, especially for those residents’ where capacity was challenged; family or friends would choose products on behalf of the resident.

Ryan and Scullion^23^ in their 2000 study of family and staff members documented that family members considered themselves to have a more significant role in caring for relatives than that acknowledged by care home personnel. The present findings have confirmed and illuminated the extent of this role in terms of supporting oral care, with care home personnel predominantly delivering “technical care” and family providing more personal social support.

The present study highlighted the significant role that family and friends have in supporting the self‐management of care home residents’ oral health problems. Care home personnel were reliant on family and friends to fulfil this role; however, the evidence suggests that this additional input can also present challenges. A Swedish study investigating family involvement in nursing home care referred to a “betweenship” where relatives are “squeezed between different obligations, interests and competing power structures among the immediate family, the nursing home as an institution and the nursing staff.”^24^ This parallels with the theme on “balancing roles and maintaining the equilibrium” stemming from our research. Other research has identified a “monitoring” role of relatives acting as a safety net “checking” that their relatives needs are being cared for and intervening when necessary.^25^ This echoes our findings, although we also observed that when residents had little or no support from family or friends, the care home personnel also undertook this “monitoring” role to help maintain the equilibrium for the resident. While relatives were not interviewed within this study, it was evident from the interviews with the residents that family and friends and their life events had an impact on the amount of support they were able to offer the resident.

In terms of oral care, care home personnel often describe themselves as having a secondary role, stepping in where the resident could not do this for themselves, or when relatives/friends were unable to provide support. Those residents who did not have relatives/friends to support them were often reliant on care home personnel to act as advocates for oral health care. This was most evident in relation to accessing dental services, as this was most commonly supported by family and friends. Interviews with care home personnel confirmed this as care home personnel also reported having difficulty in accessing dental care for residents.

Although in this study there was evidence that at least one carer, on identifying a resident's need, adjusted her professional carer role to a dual role, taking on some of the roles of family and friends. Given that this study emphasises the role that family and friends play in supporting oral health it also raises concerns for those residents without this support network. This group is at disadvantage and their oral health may be compromised.

While family and friends supported oral care, there was no indication of any evidence‐based information provided to them by either the care homes or the local dental services. Decisions about care were most often based on lay choice and advice.^26^ While studies have shown that friends and family can support care there is little evidence to indicate the best way to ensure that informal care is in line with best practice,^16^ as oral health guidance has focussed on advice for clinicians and not informal caregivers.^26^, ^27^ This research also highlights the fact that “lay” people were not always able to identify oral health conditions and their well‐intentioned actions could inadvertently be detrimental. The present study therefore suggests that there are opportunities to improve oral health by providing support for family and friends of those people who are living in care homes.

Our study found that “daughters” were the most frequently mentioned family co‐supporters of care. This resonates with the existing research base. Angelina Grigoryeva^28^ in her recent US analysis of gender division of older people parent care in sibling groups, found that daughters provided significantly more care; on average 12.3 hours a month, when compared with sons who on average provided only 5.6 hours per month. Grigoryeva also found that sons tended to care for fathers while daughters tended to care for mothers; as most participants in this study were women over the age of 85, we did not observe the latter phenomenon.

A limitation of the research in relation to exploring the family and friends perspective is that we were unable to undertake interviews with them, as this was beyond the scope of our primary study. Future work to gain greater understanding of the perspectives of family and friends and their roles in supporting oral care would be beneficial. Furthermore, only residents who were deemed to have capacity took part in this study, therefore, the views of those with more extreme cognitive impairment and dementia are not reflected. Again by talking to relatives, data on these residents could be included.

## 5 CONCLUSION

Family and friends were identified as co‐supporters of oral care and have an active role in encouraging and supporting oral care and access to treatment. Our study suggests that there are opportunities to improve oral health by providing support for family and friends of those people who are living in care, especially in relation to supplying oral care products, enabling self‐management of oral care problems and accessing dental care. Care home personnel need to actively help residents who do not have family and friends to support their oral health and care. Policies, training and services need to acknowledge and support these roles for both residents with family/friend caregivers support and more importantly for those residents without this informal support.
